# The X-ray as Applied to Dental Practice

**Published:** 1902-09-15

**Authors:** L. E. Custer

**Affiliations:** Dayton, O.


THE
### DENTAL REGISTER.

Vol. LVI. September 15, 1902. No. 9.

### THE X-RAY AS APPLIED TO DENTAL PRACTICE.

BY L. E. CUSTER, D.D.S., DAYTON, O.

Read before the Detroit Dental Society, April 7, 1902.

*Mr. Chairman and Members of the Dental Society:* —I hardly know how to begin after listening to such flattering eulogies as have been given by the President. I have been thinking while sitting here that I am in the presence of men who are experts in the use of the X-ray and also in the presence of those who have had little or no experience in this line of work; it will therefore be somewhat difficult for me to go over the ground without showing and saying some things which may be old to those who have done this work, or I may omit things which appear simple to me but which will not be thoroughly understood by those not familiar to the work. I will presume that we are here for mutual instruction and not to show what we know, so if there are any questions to be asked during the operation, do so. Very often things are omitted by the speaker which he thinks of little importance, and yet to the hearer they are of greatest importance.

In reference to the nature of the X-ray it is quite difficult at first thought to comprehend, and I think I can do no better than refer you to the chart on the wall which I have had copied from a standard work, comparing the vibrations of air with those in ether per second.

AIR VIBRATIONS PER SECOND.

| | | |
|---|---|---|
| 40,000 | vibrations, | the highest audible sound. |
| 4,000 | " | to the highest musical note. |
| 2,000 | " | to the highest soprano note. |
| 512 to 256 | " | woman's conversational voice. |
| 128 | " | man's conversational voice. |
| 16 | " | the lowest audible sound. |

ETHER VIBRATIONS PER SECOND.

| | |
|---|---|
| Trillions, | the X-rays. |
| 2,000 billions, | the actinic rays. |
| 750 billions, | the violet color. |
| 400 billions, | the red color. |
| 100 billions, | infra-red. |
| 230 millions, | the Hertz waves. |

You all understand that air is of value to us only in that it will communicate to us sound and supply us with oxygen to breathe, only in these two particulars is the air of any value to us as human beings. If an electric bell is placed under the receiver of an air pump and the air gradually exhausted, you will accordingly hear less sound, until finally when nearly all of the air is exhausted, you will hear no sound whatever, and will only know that it is ringing by seeing its vibration. Again, in ascending a mountain you will not only experience difficulty in hearing but also in receiving enough oxygen for the oxidation of the blood. In both these instances the air is of value to you only as an agency through which you hear and receive oxygen. On the other hand there is another agent and that is the ether which is of value to us in possibly five or six different ways. It is the agency of conveying to us light, heat, electricity, magnetism, X-ray and wireless telegraphy. All these are forms of ether motion. It is but natural that we should be more familiar with the air vibrations because its phenomena are more tangible to our senses, but those of the ether are also just as important although not so thoroughly understood by all. Even electricity and magnetism are not fully understood and when to-day we branch out into new discoveries it is no wonder that it seems a vague problem.

The X-ray was discovered by William C. Roentgen, of Worzburg, Germany, in November, 1895. The discovery was said to have been the result of an accident, yet it was no more than the outcome of a long and tiresome series of experiments, and Prof. Roentgen should receive all the glory which could be meted out to him, just as should every one who has studied along the line of human progress. Beginning at the invention of Crooks' tube by Prof. Crooks, in 1879, up to the investigations of Lenard, who found that the rays were transmitted to the outside of a generating tube ; it seems it was an easy thing for Roentgen to have discovered the X-ray. These rays differed very much from all other known waves, and for want of a better term, he modestly called them the X (unknown) rays. All of the above mentioned workers were very close to the goal.

The X-ray is generated within a tube which is a modification of a Crooks' tube, the ray is supposed to generate at the cathode and reflect out from the target at the annode at an angle of forty-five degrees. It has been found that if the air has been exhausted from a tube to the degree of one-tenth of an atmosphere it is a very easy matter for a current of electricity to pass from one terminal to another, much easier than if passing through the open air.

I will pass the current through about ten feet of glass tubes containing various substances to give different colors. They are so connected in series and you will observe that the current will pass through all these tubes in preference to jumping through a gap of one inch in the air.

The X-rays differ from other known ether waves in several particulars. For example, light rays may be refracted, deflected or condensed, but the X-rays can not be made to do so. The Lenard rays, which also move from a cathode and very closely resembles the X-ray, can be deflected by a magnet and also refracted while the X-ray can not.

Dr. Crooks was the first who succeeded in exhausting the air from the tubes to almost a complete vacuum, thus making possible the investigation which followed. When a current is passed through a tube of this kind the ether is thrown into such vibrations that the ray is generated within the tube. The vibrations of this ray are of an extremely high frequency and of short wave length and are supposed to be transverse. The nearer to an absolute vacuum a tube can be made the more penetrating will be the X-ray which emanates from it. It is possible to get such a high vacuum that it is impossible to generate a current of sufficiently high pressure to produce the rays.

The manner in which the current is developed to such high pressure and of comparatively small ampere strength is by three appliances: 1, the static machine; 2, the Tesla coil; 3, the induction coil.

The static machine is used extensively by physicians for the reason that they use this machine in their therapeutic treatments, but it is objectionable because of its unreliability and bulk. It is less liable to produce a burn than any other machine, but such a thing with any other appliance would be due entirely to carelessness in exposure, either too long a time, or a current of too great amperage. To sum the whole matter up, what would require a man ten minutes to do with a static machine could be done in less than a minute and better with a coil, as time and definition are both gained by the use of the coil. In dental practice this is of very great importance for the reason that our patients are sometimes placed in uncomfortable positions in order to obtain pictures.

The second method is by the Tesla coil. It is of the least value and less certain than the other forms. I would not recommend it because of its erratic action. It has the power to throw an X-ray through a two-foot brick wall and still get an X-ray picture on the other side. However this is not important; we must be able to control the current by hair's breadth almost, this is one of the necessities in our dental work. The Tesla coil is also more liable to puncture tubes than the other methods. If any of you intend to take up this work I would by all means recommend for your use the third method, the use of the plain induction coil. If you are in a city I should recommend a coil of eight or ten inches, it will probably require about thirty seconds for the longest case of exposure for dental work from a coil of this kind. But if you are practicing in a small town where a physician would be likely to use your machine, get a large coil. I would say right here that a dental practitioner is well prepared to take up X-ray work for two reasons: he is familiar with instrumentation, and also the use of electrical instruments—I would therefore advise a larger coil, one which would be capable of furnishing current sufficient to take all parts of the body so you will be of some use to your neighboring physician.

After going over the ground somewhat hurriedly in regard to the method of procedure in obtaining current for X-ray, I will now explain the two different kinds of tubes which I have already referred to and then follow with a statement in detail of the management of dental cases.

We have tubes of different vacua in X-ray work, one of low, another medium, and one of high vacuum. The effect of these tubes is a most important consideration in X-ray work. The one of low vacuum is that which would be used in nearly all dental cases. By the low vacuum is meant one which if used when looking at the hand through the fluoroscope, it will appear black and show a dark outline of the entire hand, the bones can be distinguished only by close examination. Such a tube as this I will show you by the use of the fluoroscope. I use this for nearly all dental cases, it has another advantage also, and that is, there is little danger of puncturing the tube, and it can be operated by any eight inch coil.

This fluoroscope is a screen which is covered with the salts of boro-cyanide of platinum which fluoresce in the presence of X-rays and the bones of your hand appear in contrast to the flesh because not so many rays pass through the bones as the flesh.

There are self-regulating tubes on the market but these are not necessary at all in dental work as the time for operation is so short that the tube can not be prepared before beginning.

The dentist should begin with two tubes, one of a low and the other of a medium vacuum. They are cheaper, and also in the course of time when the vacuum rises, they can be used for fluoroscopic work of thicker parts. Most tubes with regulating devices are at a high vacuum and it takes several minutes to work down to a proper condition of vacuum for dental use and probably by this time the film would have been exposed so long, it would be luck if the skiagraph were a good one. I will not go into the various methods which have been devised for regulating the vacuum.

A very important step in the production of a current for X-ray work is that of interrupting the current. There are several kinds of interruptors which have been used, but I will not mention them. Will simply show you one which is much better than any other I have seen. It consists of a jar in which there is a dilute solution of sulphuric acid in the proportion of about one to four. In the bottom of this jar is a sheet of lead which acts as one electrode. Suspended from the top and hanging into this solution is a porcelain cup of any shape, just so it is long, on the side near the bottom of this cup a small hole is made which is about the size of number fourteen gauge wire, inside of this cup is a coil of lead wire, I make a coil so as to give considerable surface, this is the other electrode. When the current passes from one of these plates to the other it decomposes the acid into its component gases, which is a most perfect insulator, this breaks the current. The solution then flows into the opening again completing the circuit. In this way, by the liquid flowing in and the repeated formation of gas we get a making and breaking of the current. There is no mechanical interrupter which acts so well as this. When I first made this, I placed the holes in the bottom of the cup, but I soon found that they filled up with gas and refused to act, but on placing them on the side of cup I have no such trouble.

The Wehnelt was the first interrupter of the kind, that is based on the electrolytic action. It consists of a glass vessel in which a lead plate forms one electrode and the platinum point the other. They are suspended in a solution of dilute sulphuric acid, the platinum point passes through the stem of a clay pipe, allowing it to extend into the solution about one-eighth of an inch. The wire in this case becomes enveloped by the gas which acts as a very high resistant for a moment and an explosion follows which affects a complete break in the current. It is very irregular in its interruptions and will also cease to work after a while. It also refuses to work when too much current is passed through it.

Caldwell invented this form which I have here, with the exception he used glass cups instead of porcelain. I found that the glass broke quite frequently and easily so I finally bought a ten-cent china cup and drilled a hole through the bottom of it and this way solved the whole difficulty. It is necessary to grind the bottom of cup as thin as possible, the thinner the porcelain the more rapid the making and breaking. The lead in this must be replenished about once a year.

I am sorry I can not show you the difference between this current when I use the old vibrator, and as it now is with this interrupter. The spark is at least ten times as great as it used to be. Dr. Price has done a great deal of work in this line and he still insists in the use of the Wehnelt interrupter, but I am sure if Dr. Price ever tries this he will agree with me that it is the best he ever used.

I will now take up the necessary step in the exposing of a patient to the rays for a skiagraph. The most frequent uses for the X-ray in dental work is for the locating of objects lodged in the root canals of teeth, locating position of unerrupted teeth, for outlining the antrum, diagnosing fractures of jaw, extent of alveolar abscesses, etc. The differentiation between hard and soft tissues is easy, still when it comes to the infiltration of the antrum which differs but little from the surrounding tissue, it can still be done. I have a number of photographs taken of cases which have been presented me in practice and at meetings of this kind. This is the first case and most interesting: This patient had been wearing an upper and lower plate for eight years and had had two operations performed in the roof of the mouth for abscess, the operators went at it blindly and could not locate the cause. This picture, my first attempt, shows the presence of a foreign body in the roof of the mouth. I took out the root, being able to locate it from this picture. Here is a picture of a case of malignant sarcoma; was able to operate and also to save the molar on each side.

Dr. Taft: Is there anything to aid you in the diagnosis of tumors?

Dr. Custer: Only as to extent. No characteristics whatever. Nearly all these photographs which I have here are the results of my first attempt at photographing and I might add right here that the greatest credit in the world is due to Dr. Price for the working out of photographic detail in X-ray work, we owe everything in this line to him.

Here are several pictures taken of different cases, impacted third molars, retarded erruption of molar, etc., in which you can see very clearly the distinct outline of the antrum. I will pass these pictures around.

Now, if there are any particular cases here, I will be glad to expose the patient for radiograph as I have several prepared films. These are the regular kodak films but especially prepared with three coatings, as plates prepared in this way give better details. Seed & Co. prepare these films which come in sheets and can be cut into the shape desired, and then enclosed according to whatever method you desire. I enclose these films between two thicknesses of black paper, the film is placed sensitive side down upon a piece of paper larger than the film which is then turned over, on this is then placed another piece of black paper and turned over in the same manner. Prepared in this way they are flexible and can be adapted to any part of the mouth. Dr. Price prepares his films by first covering with a piece of parafine paper and then this is placed between a piece of black unvulcanized rubber twice as large as the film, this being placed on one half and the other half turned over and the edges of rubber pinched together, this makes a light and water-proof covering and is very good in prolonged exposures.

I have never found long operations necessary and have never destroyed a film by my method. All this work, of course, is done in a dark room.

(The first case which was presented to Dr. Custer for a picture was a patient of Dr. Oakman's, where there were no bicuspids on the right side of the mouth, and the cuspid was in the position of the second bicuspid.)

It is necessary in all such cases to have the area to be photographed as near perpendicular to the target as possible, in placing the film in the mouth first conform it to the palate with the fingers and keep the head as still as possible and about fifteen inches from the tube. The length of time for the exposure will depend upon the condition of the tooth, if I had a tube of proper vacuum it could be done in two or three seconds, it will however take me about ten seconds in this case. I will make two exposures so as to be sure of a negative. These films are covered with two layers of black paper.

The second case presented was one of diseased antrum. Dr. Oakman placed in a piece of fused lead wire so as to locate the antrum. In such cases as this it is hard to get a picture of the antrum as it is much the same in density as the surrounding tissue. It is of particular value when a foreign body is lodged within the antrum.

### DISCUSSION.

Dr. Hickey: *Mr. President and Gentlemen:*—I am very diffident in getting up to say anything about the X-ray after listening to the remarks of a past-master in the art. I think that perhaps Dr. Cook and myself are in position to appreciate the work which the Doctor has done better than some of the gentlemen present. In taking up X-ray work within the last few years I discovered that I was working in a field where there was very little literature, and a good deal of the stray literature which appeared in the journals was contradictory, and I might add that the knowledge which I have obtained has had to be worked out by perseverance and hard work. The technical difficulties in X-ray work, management of the large coil, interrupter, etc. which I have experienced, cause me to appreciate more fully the remarks of Dr. Custer about the old-fashioned interrupter, etc. The mastery of the photographic technique is one which also requires perseverance and application.

In regard to the application of the X-ray in medicine there is a greater field there than in the application of the X-ray in surgery. When first brought to the attention of the medical profession they thought that the X-ray would be applicable simply in the diagnosis of broken bones and the location of foreign bodies, but work which has been done within the last three or four years shows that internal medicine can expect great aid from the X-ray. With the perfection of the apparatus and careful use of the proper technique we are able to get a great deal of assistance from the X-ray in diagnosing diseases of the thorax, for example, you can outline most brilliantly the position of the diaphragm, can tell if its excursion was regular and also the extent of its excursion upward and downward, also in beginning cases of tuberculosis it will show a limited excursion of the diaphragm on the affected side.

In cases of pleurisy you may see the level of the fluid as correctly and much more so than you can ascertain by the tapping of the chest. For example, it is much easier to tell how much water there is in a barrel by seeing into it than it is by tapping from the outside. The position of the heart can be correctly made out. It is very difficult to do so by the old-fashioned method of osculation. There is no way by which you can diagnose aneurisms as correctly as with the X-ray.

In regard to the therapeutic use, this is a field in which a great deal of experimental work is being done at the present time. It has been proven by cases treated in various parts of the world that it is a correct treatment for superficial lupis. Whether it will prove as beneficial as carcinoma, as some claim, I think it is a question of demonstration.

DR. COOK: *Mr. President and Gentlemen:*—I have been very much pleased by Dr. Custer's demonstration and his ideas put forward, it seems that there is very little left for me to say. So far the operative technique of the X-ray work has been in question, as stated by different operators, in so much that the tubes used are never constant. The working distance also is not distinctly defined. The exposure has been by some writers stated as a certain length of time and by others for the same distance as a different length of time. This is because they have been using tubes of different vacuua. This is what has caused this difference in their statements.

Its field in medicine is simply opening up, at the present time there are different X-ray specialists who are reporting wonderful results in the cure of carcinoma and sarcoma, but it will be some time before definite knowledge can be obtained as to any recurrence of the disease.

I have been very much interested in the Doctor's speaking of the use to which the X-ray can be put in dental surgery, and it seems to me in certain cases of unerrupted teeth it would operate very satisfactorily It would also aid them in correcting some deformity in taking care of the teeth with a view or idea of some future difficulty. I wish to compliment the Doctor upon his able demonstration.

